# A reward self-bias leads to more optimal foraging for ourselves than others

**DOI:** 10.1038/s41598-024-69452-x

**Published:** 2024-11-05

**Authors:** Luis Sebastian Contreras-Huerta, M. Andrea Pisauro, Svenja Küchenhoff, Arno Gekiere, Campbell Le Heron, Patricia L. Lockwood, Matthew A. J. Apps

**Affiliations:** 1 Department of Experimental Psychology, University of Oxford, Oxford, Oxford OX1 3PH UK; 2 Wellcome Centre for Integrative Neuroimaging, University of Oxford, FMRIB, John Radcliffe Hospital, Oxford, OX3 9DU UK; 3 Centre for Human Brain Health, School of Psychology, University of Birmingham, Birmingham, B15 2TT UK; 4 Institute for Mental Health, School of Psychology, University of Birmingham, Birmingham, B15 2TT UK; 5 Center for Social and Cognitive Neuroscience (CSCN), School of Psychology, Universidad Adolfo Ibáñez, Viña del Mar, Chile; 6 Department of Medicine, University of Otago, Christchurch, New Zealand; 7 Christ Church, University of Oxford, Oxford, OX1 1DP UK; 8 School of Psychology, University of Plymouth, Plymouth, UK; 9 Department of Experimental Psychology, Ghent University, Ghent, Belgium; 10 New Zealand Brain Research Institute, Christchurch, New Zealand; 11 Center of Social Conflict and Cohesion Studies, Santiago, Chile

**Keywords:** Psychology, Human behaviour

## Abstract

People are self-biased for rewards. We place a higher value on rewards if we receive them than if other people do. However, existing work has ignored one of the most powerful theorems from behavioural ecology of how animals seek resources in everyday life, the Marginal Value Theorem (MVT), which accounts for optimal behaviour for maximising resources intake rate. Does this self-bias help humans maximise rewards when foraging for their own benefit compared to foraging for the benefit of others? Participants had to decide when to leave patches where reward intake was gradually depleting, in environments with different average reward rates. Half of the time participants foraged for themselves, and in the other half they collected rewards for an anonymous stranger. The optimal MVT derived solution states people should leave when the instantaneous reward intake in a patch equals the average rate in an environment. Across two studies, people were more optimal when foraging for self, showing a reduced sensitivity to instantaneous rewards when foraging for other. Autistic traits were linked to reduced sensitivity to reward rates when foraging for self but not for other. These results highlight that the self-bias may be adaptive, helping people maximise reward intake.

## Introduction

Humans are self-biased when it comes to rewards. We do not value and are not as motivated to obtain rewards for other people as much as rewards we can get ourselves^1,2^. Decades of experiments have examined this reward self-bias, typically in experiments where people make binary choices between options associated with an immediate reward that is delivered to either self or other^3^. Yet, despite the important advances made using such paradigms, binary scenarios may not reflect the types of decision problems faced by animals, nor by our hunter-gatherer ancestors^3,4^. Moreover, these studies do not consider whether people’s decisions to prioritise their own rewards actually lead to optimal behaviour in terms of maximizing reward intake in different environments. Therefore, it remains unclear whether the self-bias enhances our ability to efficiently collect rewards.

Prosocial behaviour is highly prevalent among non-human animals. Many species who live in groups forage for resources and share them with conspecifics^5,6^. Humans are a similarly prosocial species—we often act to benefit others^7^. Human hunter-gatherers, both in the past and present, employ similar methods for searching and collecting resources as other animals, and these resources are commonly shared within their communities^8–11^. The effectiveness of these hunter-gatherer strategies in facilitating resource-sharing aligns with human prosocial tendencies, which are often rooted in reciprocity and cooperation and extend beyond immediate family ties^12^. While there is evidence of humans demonstrating prosocial motivation in various scenarios, it remains unclear whether foragers obtain rewards as efficiently for others as for themselves. Notably, the presence of a self-bias observed in previous literature could imply a bias towards collecting resources for oneself relative to others in optimal foraging behaviour, given the potentially lower or no direct energy gain when foraging for others. However, persistent prosocial motivation in other contexts challenges this assumption^12–14^. Consequently, our study aims to unravel how humans make foraging decisions when collecting rewards for others and whether this differs from their decisions when collecting rewards for themselves.

To examine whether the self-bias for rewards results in humans making foraging decisions that increase reward intake for ourselves than when foraging for others, here we use a powerful theorem from behavioural ecology^15^ that provides an optimal solution to a key decision problem faced by many species. Many species must decide when to leave a current location to collect resources in another—the patch-leaving problem. Resources, such as food, are not located everywhere evenly, e.g. some bushes (patches) have more berries in than others. Moreover, some environments are sparser than others e.g. some have fewer bushes with lots of berries in than others. Thus, to maximise reward intake when making patch-leaving decisions, one needs to consider both how good a patch is, and how rich the environment is.

This problem has an optimal solution, formalised within marginal value theorem (MVT^15^)—the optimal forager should leave the current patch when the rate of reward they are getting (foreground reward rate, FRR) falls to the same level as the average reward rate that can be obtained in the environment (background reward rate, BRR). Crucially, this means statistically that they should both show independent effects on behaviour and there should be no interaction of their effects. I.e., the effect of changing the BRR should be the same on all patches. Thus, an agent should stay longer in high yield patches with a lot of resources than low yield patches. But also, the agent should stay longer in all patches in poor environments with low BRR, where patches are far apart or most of them are low yield, than in richer environments with high BRR. Many species conform to these principles and are close to optimal when foraging in the wild, including human nomadic tribes^16–24^.

Recently, experimental paradigms inspired by the patch-leaving problem have been used in humans, showing that, broadly speaking, choices to leave a patch conforms to the principles of MVT^25,26^. However, people depart from optimal leaving times, and variability in people’s sensitivity to information in these tasks has been associated with individual differences in psychological variables^27,28^. Yet, it is unclear whether people forage optimally and maximise reward intake when making leaving decisions that prosocially benefit another person. In strict terms, MVT does not predict prosocial behaviour in a foraging context, as the forager does not derive any direct energy intake from such behaviour. Nevertheless, given that individuals still engage in communal foraging^10,11,29^, display prosocial behaviour in various contexts^2,7,30–32^, possibly rooted in evolutionary mechanisms^13,33^, and exhibit an ability to encode rewards of others^34–38^, influencing motivation^34,39,40^, it becomes conceivable that they might adhere to prosocial foraging in line with MVT principles.

Our study seeks to explore whether the reward self-bias is evident in an MVT-like context, examining whether individuals show a bias toward themselves in processing both FRR and BRR. The primary aim of our study is to compare foraging behaviour when collecting rewards for others versus when collecting for themselves, and to contrast these behaviours with MVT principles to assess their optimality. However, previous studies have shown that while animals and humans generally follow MVT principles, they also exhibit an overstay bias, leaving patches later than what MVT predicts^16,26,41^. Despite this, both humans and other animals tend to leave sooner patches in rich environments compared to poor ones, and sooner from low-yield patches relative to high-yield ones. Therefore, our study also aims to test for optimality by examining these effects while correcting for the well-documented overstay bias. This approach allows us to understand foraging behaviour beyond the strict application of the MVT.

Here, we designed a task based on MVT to examine whether people make patch-leaving decisions differently when collecting rewards for self or for an anonymous other. In two studies, participants collected rewards from patches, in different environments, where the rate of obtaining rewards continuously depleted. Participants spent five-minute periods collecting rewards from patches in environments with different average reward rates. There were two types of patches, starting with either high or low yields (i.e. creating high or low FRR patches). In addition, there were two types of environments, which could be either rich (high BRR) or poor (low BRR). Crucially, half of the time participants spent in environments where the reward was collected for themselves, while the other half they collected for another person. The participants’ task was to decide when to leave the current patch and spend some time travelling to the next one while not receiving any rewards.

Using this design, we could test the hypothesis whether the reward self-bias leads to people to be more sensitive to FRR or BRR when foraging for oneself than for others. If the self-bias is adaptive, then people would show a difference in patch-leaving times between self and other, and would be closer to the optimal leaving times according to MVT. Moreover, in exploratory analyses we examined whether traits that have been associated with reward-based behaviour for self and others such as apathy^27,42^, autism^43^ and empathy^42^ modulate foraging behaviour across FRR and BRR sensitivity for self or other. Different studies have shown that, for instance, behavioural apathy is linked to a decrease in motivation to obtain self rewards, while social and emotional apathy have been associated specifically to less prosocial motivation^42,44,45^. Likewise, autistic traits have been suggested to be involved in disrupted reward representation for both self and others^46,47^, which could misguide their behaviour. Finally, empathy, understood as representing others’ affective states, has been widely found to be linked to motivation to benefit others^31,42,48–50^. Therefore, in this study, we aimed to test whether these traits can modulate foraging behaviour for self and other, specifically considering different reward information such as FRR and BRR. Identifying specificity between self and other, and between FRR and BRR, could shed light on the mechanisms underlying motivational aspects of these traits.

## Material and methods

### Participants

The study was approved by the University of Oxford ethics committee (R55988/RE001), and followed the Helsinki Declaration of 1964. All participants gave written, informed consent at the beginning of the study. Volunteers were recruited through the University of Oxford participant databases, online platforms where students from the university and the local Oxford community can subscribe to receive information about various studies across the university. Subsequently, individuals can voluntarily choose to participate in studies based on their interests and availability. They gave informed consent, and they were paid for their time (£8 per hour, plus a bonus £4). For study 1, 45 participants were recruited. From this sample, one participant did not complete the entire task, one suffered technical issues while completing the task, two expressed doubts about the social manipulation, and one did not show engagement when collecting rewards for others (leaving times < 500 ms). This resulted in a total sample of 40 (age m = 24.8, s.d. = 5.2, 26 females).

Our design has the challenge that different participants will have different number of trials per condition, as our main behavioural index is patch leaving times, which varies across subjects. To ensure sufficient power to observe similar results in study 2 as in study 1, we calculated the necessary sample size before conducting study 2. We estimated the sample size for study 2 based on the mean and standard deviation of the number of trials per condition in study 1, together with the random intercept and the fixed effects of the mixed models (MM) on leaving times (see Statistical Analyses below). With these parameters, leaving times were simulated 2000 times: 1000 times for 25 participants, and 1000 times for 30. For each simulation, we ran a MM for main and interaction effects. Power was defined as the number of times the effects found in study 1 were significant (*p* < 0.05) divided by the total number of simulations. Based on these simulations, a sample size between 25 and 30 participants was sufficient to capture the main and interaction effects from Study 1 with a power of > 0.8. Thus, data were collected from 31 volunteers for study 2. However, two participants failed to follow instructions, with leaving times being either very short (< 500 ms) or long (> 3 s.d. from the sample average) and thus were excluded. Therefore, the final sample in study 2 was 29 (age m = 24.7, s.d. = 5.0, 16 females).

### General procedure

At the beginning of the experimental session**,** participants were told that they were one of two participants who would perform two different decision-making tasks. After participants answered some demographic questions, they were randomly assigned one of the potential tasks, following a strict protocol used in previous studies^45^ (see Supplementary Information, SI). In reality, participants always completed the self-other foraging task, as the other participant was a confederate. The confederate was part of the experimental team and acted as the second participant. Participants were informed that they would be engaged in a task wherein they would collect rewards for both themselves and for another participant. They were explicitly told that what they earned for each beneficiary was independent of the other, and that their decisions would remain confidential and anonymous. Importantly, participants were explicitly informed that the total reward collected for the other person would only be disclosed to the experimenter responsible for testing the other participant. This measure was implemented to prevent any opportunities for participants to compare their performance for themselves and for the other person at the conclusion of the experiment. Following the completion of the task, participants were asked to respond to self-report scales and debriefing questions regarding the social manipulation setting.

### The self-other foraging task (SOFT).

#### Experimental design

**Figure 1:**
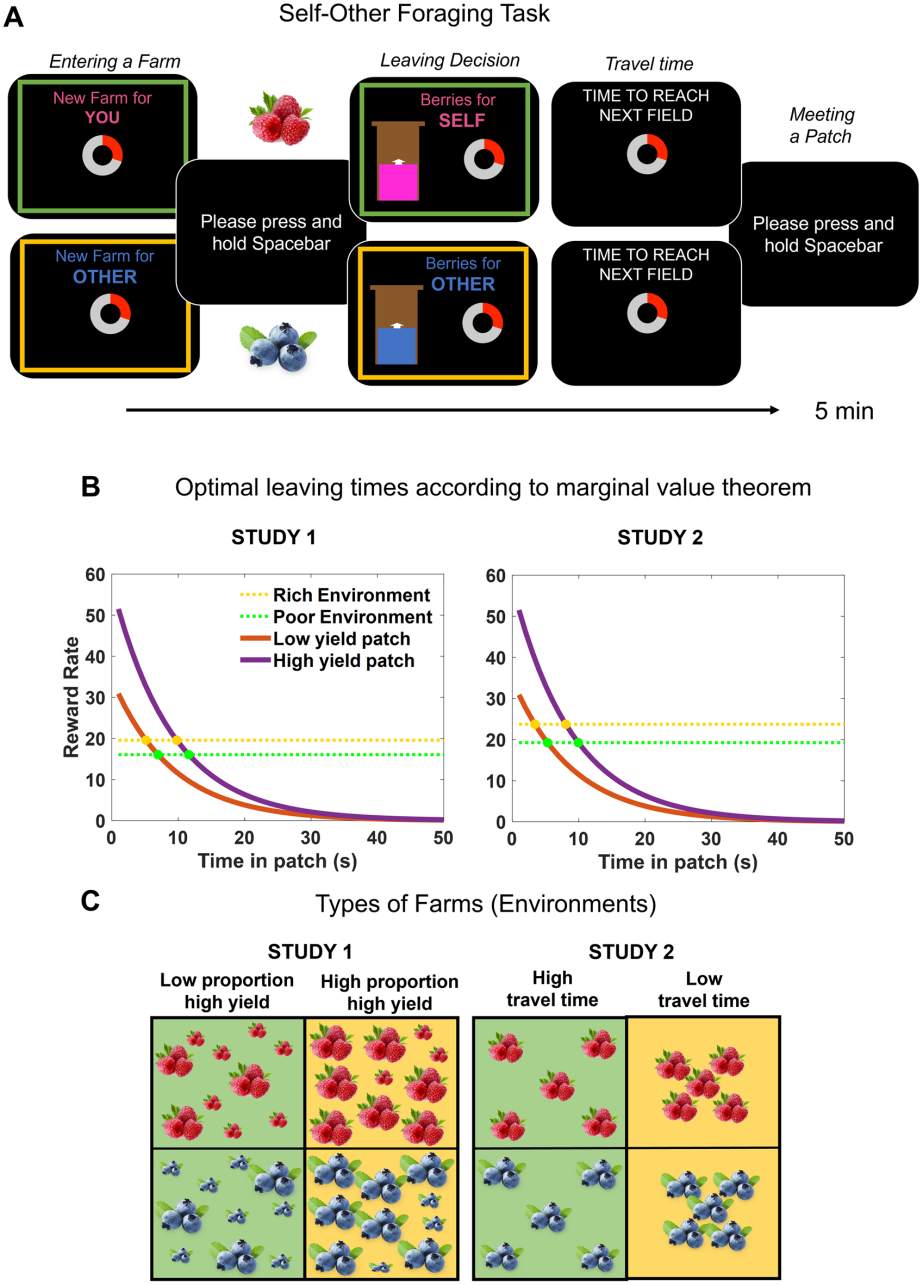
Task and design. (**A**) Self-Other Foraging Task (SOFT). Participants made decisions of when to leave patches (field) in different environments (farms). When entering a farm, a screen indicating the farm type (gold or green outline square indicating rich or poor environment) and the beneficiary (raspberry for self or blueberry for other) appeared (*Entering a farm* screen). Participants then spent 5 min in the farm, choosing when to leave a current field in which berries (reward) were collected at an exponentially decreasing rate. The (foreground) reward rate was illustrated by a brown basket being continuously filled by either raspberries or blueberries (*Leaving decision* screen). Once participants decided to leave, they incurred a travel time cost during which they did not collect rewards, represented by a clock ticking down (*Travel time* screen). Finally, participants encountered again a patch, which will give the rewards once the press the spacebar (*Meeting patch*). The, sequence starts again until 5 min have passed. After this, participants entered into a new farm, getting the *Entering a farm* screen again. (**B**) Optimal leaving times. Continuously decreasing foreground reward rate functions–that dictated the speed the basket was being filled during the task–for the high and low yield patch (purple and orange lines, respectively), as well as background reward rates for rich and poor environments (gold and green dotted line respectively). Where the lines intersect is the optimal leaving time for each patch in each environment that will maximise reward intake. (**C**) Representation of background reward manipulation. Participants visited four types of farms (2 beneficiaries: raspberry/self, blueberry/other) × 2 environments: rich/gold, green/poor). The gold farm had a higher background reward rate than the green farm. In study 1, the rich environment had a higher proportion of high yield (represented by big berries in the figure), and a lower proportion of low yield patches (represented by small berries in the figure), while the opposite was true for the poor environment. In study 2, the rich environment had shorter travel times (2.8 s) than the poor environment (5 s). Thus, patches in the gold environment were ‘closer’ to each other compared with the green farm, but had the same proportion of high and low yield patches.

Participants completed a computer-based task that emulates a patch-leaving problem (Fig. 1A), deciding when to leave from the current patch to the next, designed following the principles of MVT (Fig. 1B, see SI). The SOFT was framed as a farming game, where participants had to collect berries (reward) on sequentially encountered fields (patches) within a certain farm (environment). Participants were told that the berries they accumulated in the experiment would serve as a monetary bonus for themselves and the other participant. This bonus, that was up to £6, would be added to the baseline money given to both participants as part of the experiment. In reality, however, all participants received £4 for their performances. There were two berry farms: raspberry and blueberry, where they collected berries to obtain money for themselves and for others, respectively. Crucially, there were also two farms/environments BRR. This was depicted to participants with coloured borders on the screen. In gold farms, the BRR was higher than in green farms. Participants were not told exactly the BRR for each type of environment as reward was in arbitrary units and delivered in a continuous rate (see below). Participants, therefore, had to estimate the BRR from the reward they experience in each patch, the encounters with each patch, and the travel time between patches, to behave optimally. The BRR was manipulated differently for study 1 and 2 (see below, Fig. 1C).

In a block design, participants visited each of the four farms—two BRR (gold/green) x two berries (raspberries/blueberries)—for five minutes, where they collected berries from the fields they encountered. They could leave the patch to travel to a new one at any time. However, moving from one patch to another had a time–cost associated, a lapse of time where participants did not collect any reward. The number of patches participants could potentially visit was unlimited but constrained by the amount of time participants could spend in each specific farm. Participants were told that their goal was to decide when to leave the current patch to move to the next one, based on the provided information. By giving this instruction, we avoided influencing participants' decisions with a specific strategy that might be the same for both self and others (e.g., "maximize reward intake"). This allowed participants the freedom to choose their own strategy for each condition, allowing us to observe whether MVT principles would naturally emerge from their behaviour.

When a participant was in a patch, its instantaneous FRR decreased exponentially. There were two types of patches (Fig. 1B), defined by the following patch reward function:

1 $$g\left(T\right)=S*{e}^{-0.11* T}$$

where *g* is the FRR after *T* seconds. Patches varied in the scaling factor of the reward function S, with high yield patches having an S value of 57.5, and low yield patches a value of 34.5. This defined the starting amount of reward in the patch. Thus, in high yield patches, rewards had a higher initial rate than the low yield patches, but decayed in the same manner, meaning that the rate would remain higher for longer than in low yield patches, and therefore, it was beneficial to stay longer. The FRR was represented by a basket continuously filled with berries while participants were in the patch. The level of the berries in the basket was proportional to the integral of the patch reward function between time zero and T, being updated with a frequency of 20 Hz. Even though participants knew that there were two patches across the experiment, they were not explicitly told the FRR of the patch they were collecting berries from. Rather, they had to deduce it from the rate at which the basket was getting filled on each trial.

Using MVT (see SI), we designed studies 1 and 2 with optimal leaving times calculated in advance based on the parameters specific to each experiment (Fig. 1B). Thus, longer patch residency time is predicted for high than low yield patches regardless of the environment, as the former reach the BRR later than the latter. This effect (difference in optimal leaving times between high and low yield patches) was set at 5 s (rounded) across studies 1 and 2. Furthermore, MVT predicts a longer residency time in the poor/green environment than in the rich/gold environment irrespective of the patch. The environment effect (difference in optimal leaving times between poor and rich environment) was set in 2 s (rounded) across studies. Importantly, notice that the reward rate at the time of leaving is the same for high and low yield patches within an environment, as at the optimal time of leaving a patch its reward rate equals the reward rate of the environment. Therefore, for statistical analyses, the main indexes used were leaving times and reward rate at time of leaving, as they reflect the behavioural outcomes in a patch-leaving problem.

#### Procedure

After participants were provided instructions and performed practice trials for the four types of farms, they started the SOFT. Instructions were delivered by the experimenter following a power point presentation illustrating the different aspects of the study. General verbal instructions prior the practice trials can be found in the Supplementary Methods.

Each task block had a duration of 20 min during which participants visited the four types of farms (raspberry/green, raspberry/gold, blueberry/green and blueberry/gold) in a random order. The introduction of a random order for the farms was implemented to prevent potential biases in participants' performance across conditions, such as treating the first farms as practice and the last ones as the main task. In line with this consideration, the avoidance of separating self and other blocks ensured that participants encountered each type of farm within each block, further minimising any systematic order-related effects. This can be reflected in participants’ leaving time per each block in Supplementary Figure S1.

Each farm started with a screen lasting three seconds giving information about the richness of the farm (gold/green) and the type of berries (raspberry-self/blueberry-other, Fig. 1A). This screen only appeared once when entering a farm. Participants then were prompted to press and hold the spacebar to automatically collect berries. Until participants released the spacebar, they were continuously collecting berries determined by the yield of the patch. The amount and type of berries collected per patch/field was shown by a continuously growing pink (raspberry) or blue (blueberry) bar that represented a continuously growing pile of berries in the participant’s basket. The rate of berries being collected slowed down exponentially as the patches resources depleted. When the participant decided to leave the field and released the spacebar, they had a variable time cost of a few seconds in which they *‘approached to the next field*’. During this travelling time, a screen with a timer was displayed. After the timer had run down, a new screen told participants that they now had arrived at the next field and asked them to press and hold the space bar until they wished to leave it. Participants did this during the five minutes that a farm lasted. After this time, a screen lasting three seconds informed them that the time had ended, followed by the screen informing them about the new fam richness and the beneficiary of the berries. Participants then entered the new farm and repeated the procedures outlined above.

#### Study 1–BRR manipulation by patch distribution

Participants completed two blocks (visiting each of the four types of farms twice) of the experiment, having a short break in between. The BRR of the farms was manipulated by varying the distribution of high and low yield patches (Fig. 1C). For the rich/gold farm, 70% of the patches were high yield, and 30% low yield. The opposite was true for the poor/green farm. In both blocks, the travel time was fixed at six seconds. The order of the patches within a farm was pseudorandomised such that in every series of 10 patches, seven were of one type and three of the other, with these three patches distributed such that no more than two appeared in a series of five patches. Patches were then randomised under these constraints.

#### Study 2–BRR manipulation by travel time

Participants completed three blocks of the experiment (visiting each of the four types of farms three times), with short breaks after the first and second blocks. Unlike study 1, the BRR was defined by the duration of the travel time associated with moving from one patch to another. Thus, in the rich/gold farm, the time needed to find another field was 2.8 s, while in the poor/green farm the travel time was fixed at 5 s. Differences in travel time were framed as distance between fields, i.e. fields in gold farms were closer to each other compared with those in green farms. Patches were equally distributed across farms in a pseudorandomised order, such that in every series of 10 patches, half were high, and half were low yield patches in a random sequence. Note that the reduction in travel times increases the BRR of study 2 relative to study 1 farms, predicting, according to MVT, lower patch residency times (Fig. 1B).

### Self-report measures

*Autism Spectrum Quotient, AQ-10*^51^*:* The AQ-10 is a 10-items self-report measure where people answer their agreement with statements. Total scores range from 0 to 10, with a score of 6 suggesting clinical levels of autistic traits.

*Apathy-Motivation Index, AMI*^52^: The AMI is an 18-item scale where people indicate their level of agreement with each item. It comprises three subscales/domains of apathy: behavioural activation, social motivation and emotional sensitivity.

*Questionnaire of Cognitive and Affective Empathy, QCAE*^53^: The QCAE is a 31-items self-report scale where people must say how much they agree with each sentence. It measures five dimensions of empathy, which can be grouped into two broader factors—cognitive and affective empathy.

### Statistical analyses

Linear mixed-effects models were applied as the primary analysis tool using *lmer* function in R, allowing us to account for differences between conditions in the number of trials^54,55^. In this model, for a trial *i,* the continuous variable patch leaving time *LT* is predicted by the fixed effects of patch yield *P*, environment richness *E* and beneficiary *B*, and their interactions, such that:

2 $${LT}_{i}= {\beta }_{0j[i]}+ {\beta }_{1j[i]}{P}_{i}+{\beta }_{2j[i]}{E}_{i}+{\beta }_{3}{B}_{i}+ {\beta }_{4}{P}_{i}{B}_{i}+ {\beta }_{5}{E}_{i}{B}_{i}+ {\beta }_{6}{E}_{i}{P}_{i}+{\beta }_{7}{P}_{i}{E}_{i}{B}_{i}$$

P, E and B were factor, binary variables. This model has a random intercept clustered in participants *j*, as participants have different number of trials per condition depending on how long they stay in each patch. Random slopes for patch and environment were also included as previous studies have shown that participants vary in their sensitivities to FRR and BRR^25,26^, but there is no interaction between them, as predicted by MVT^15,26^. Comparing the AIC of a model with only random-intercept or with random-slopes added reveals that the latter outperforms the former (see SI, Table S1). To avoid biasing effects of outlying data (implausibly early or late leaving times), we excluded trials with leaving times above 3 standard deviations from the mean of a subject, and below 500 ms prior to performing the analyses (< 0.8% of trials on average across studies). This exclusion aimed to skip those decisions where people did not track or neglected the reward intake. The total number of trials per beneficiary condition was similar on average in both study 1 (self: mean = 61.2, SD = 17.7; other: mean = 64.1, SD = 18.4) and study 2 (self: mean = 123.8, SD = 32.6; other: mean = 128.9, SD = 37.9). Finally, post-hoc analyses were performed using the *emmeans* package in *R.*

We also examined how participants made decisions in relation with the optimal solution predicted by MVT, testing their average leaving times for each condition against the MVT prediction using one-sample t-tests. However, prior studies have shown that people are suboptimal and tend to overstay in patches^25,26^. To account for this bias, we also calculated the FRR and BRR effects for self and other in each participant, defined as the difference of the average leaving times between high and low yield patches, and between poor and rich environments, respectively. We tested whether this difference was significantly equivalent to the optimal effect for FRR and BRR predicted by MVT, using equivalence tests implemented in *R* in the *TOSTER* package^56,57^. We fixed upper and lower bounds on effect size Cohen’s d = 0.5 and an alpha of 0.05, following previous studies with similar sample size^26^. We also used paired t-tests to compare the FRR and BRR effects between self and other.

Finally, we also performed a similar model to Eq. (2) to predict reward rate at the time of leaving instead of LT, aiming to test for optimality in self and other, such that:

3 $${RR}_{i}= {\beta }_{0j[i]}+ {\beta }_{1j[i]}{P}_{i}+{\beta }_{2j[i]}{E}_{i}+{\beta }_{3}{B}_{i}+ {\beta }_{4}{P}_{i}{B}_{i}+ {\beta }_{5}{E}_{i}{B}_{i}+ {\beta }_{6}{E}_{i}{P}_{i}+{\beta }_{7}{P}_{i}{E}_{i}{B}_{i}$$

where the continuous variable reward rate of a patch at time of leaving *RR* is predicted by the binary, nominal variables of patch yield P, environment richness E and beneficiary B, and their interactions. As Eq. (1), a random intercept clustered in participants j was included, as well as random slopes for patch and environment. In both LT and RR models, we included interaction main effects of P*E because, even though mathematically and theoretically there should not be an interaction effect, people might exhibit suboptimal behaviour, which could be explained by ignoring the independent effects of FRR and BRR. Furthermore, note that FRR here corresponds to the initial FRR on foragers’ leaving time, which is S from Eq. (1). Thus, FRR depend on the initial yield of each type of patch.

For correlations between leaving times and the self-report measures, Pearson correlations were used, except for those involving the AQ-10, where Spearman-rank tests were performed instead, as this scale showed very skewed distribution (common in psychiatric scales). Before testing for correlations, FRR and BRR effects (differences in leaving times between high and low yield patches, and poor and rich environments, respectively) for self and other in study 1 and 2 were z-scored. Scores for the AQ-10, the three subscales of the AMI, and the two dimensions of the QCAE, were also z-scored per study. Studies 1 and 2 samples were taken together in these correlations (n = 69). Participants who did not pass a catch question in the surveys were excluded from this analysis (n = 6). The six self-report scores were then correlated with FRR and BRR effects for self and other. P-values were corrected for multiple comparisons across the six self-report measures using false discovery rate (FDR^58^). Two-tailed tests were used for all analyses.

Crucially, the primary behavioural indices for our study were leaving times and the reward rate at the time of leaving a patch. It is important to note that differences in leaving times may not necessarily result in significant variations in the total reward accrued. This is further emphasised by the fact that reward was measured in arbitrary units, rendering the latter index less sensitive. Given that participants tended to overstay in patches, the gain in reward over time was minimal, meaning that higher differences in reward occurred early in the patch. Nonetheless, we have included the total reward accrued in the Supplementary Results for comprehensive reporting.

## Results

### Study 1

#### Foreground but not background reward rate sensitivity differs when foraging for self or other

**Figure 2:**
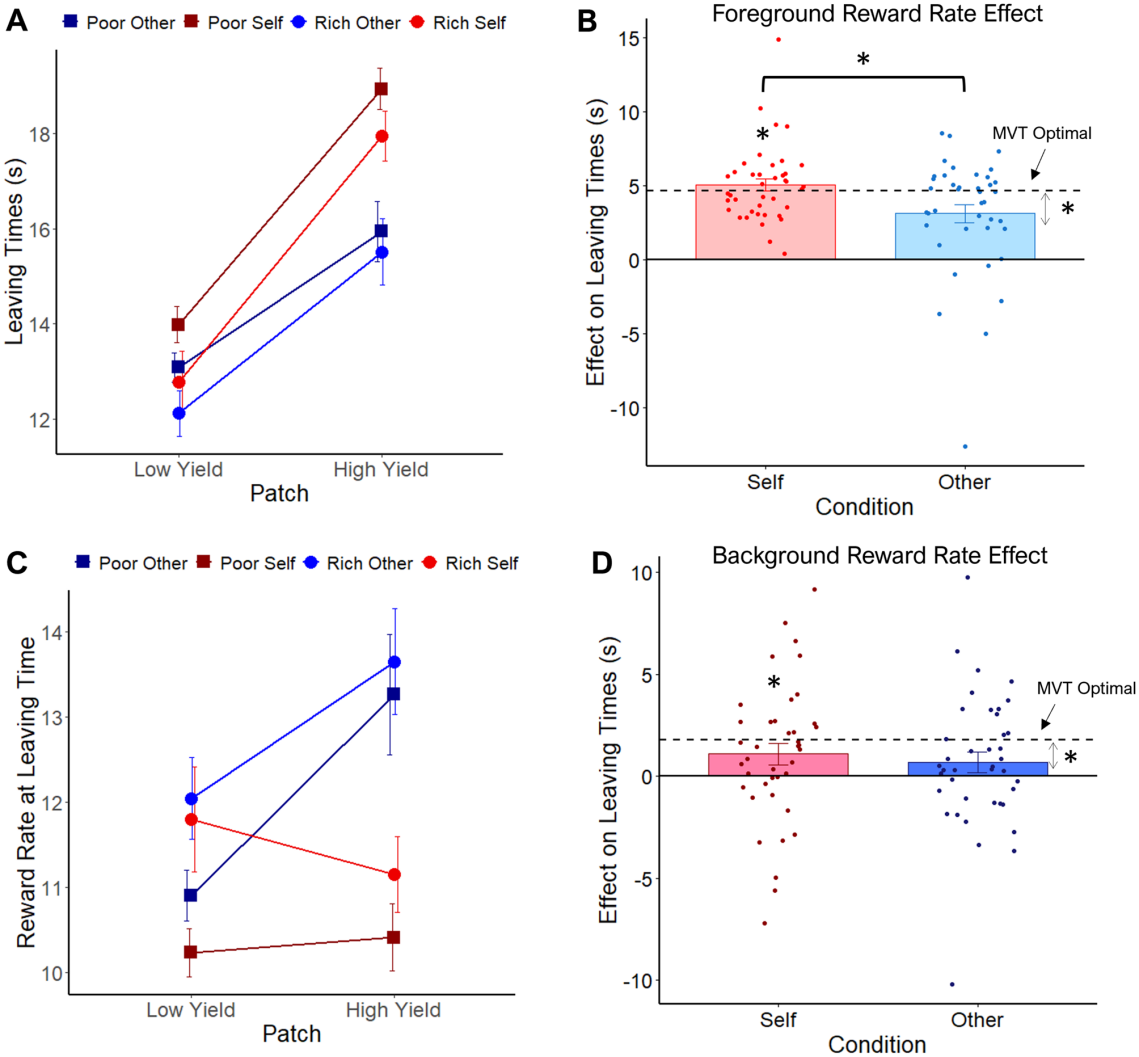
Foreground and background results of study 1. (**A**) Mean leaving times (y-axis) for each condition in the SOFT. Red lines correspond to self environments while blue lines are other environments, including rich (lighter circles) and poor (darker squares). As hypothesised, participants stayed longer in high than low yield patches (x-axis), following MVT predictions. This effect was stronger when collecting rewards for self than other. Participants left the rich environment earlier than the poor one, effect not modulated by the beneficiary of the reward. (**B**) Foreground reward rate effects on leaving times. Mean leaving times were subtracted between high and low yield patches and compared with the optimal predictions according to MVT. Participants showed foreground reward rate effect (y-axis) following MVT predictions (dotted line) only for self but not for other (equivalence test), which significantly deviated from optimal. Also, foreground reward rate effect was significantly different between self and other. (**C**) MVT predicts that the foreground reward rate (y-axis) at which participants choose to leave should not vary between patches (i.e. the lines should be flat on the graph), only changing as a function of the environment. Participants showed this effect only for self, but not for other. (**D**) Background reward rate effects on leaving times. Mean leaving times were subtracted between poor and rich environments and compared with optimal predictions. Participants followed MVT predictions in background reward rate effect only for self but not for other (which significantly deviated from optimal), although they were not significantly different from each other. *p < 0.05. Error bars depict s.e.m. in B and D, and within-subject s.e.m. in A and C.

According to MVT, FRR and BRR should have independent effects on leaving times—participants should leave earlier from patches in rich compared to poor environments, and from low vs high yield patches across environments. We tested whether participants were equally sensitive to FRR and BRR when they collected rewards for others compared with themselves through MMs predicting participants’ leaving times by type of patch, environment, beneficiary, and possible interactions. We found a patch x beneficiary interaction (t_4898.84_ = − 3.54, *p* < 0.001, Fig. 2A**;** see SI, Table S2), indicating that patch sensitivity changed depending on who was the beneficiary of the reward. Post-hoc analyses revealed that the difference in leaving times between high and low yield patches, although significant when deciding on both self (t_49.9_ = − 11.76, *p* < 0.001) and other (t_48.8_ = − 8.24, *p* < 0.001) trials, was significantly larger when collecting rewards for self than other (t_4901_ = − 5.35, *p* < 0.001). Furthermore, leaving times for high (t_4895_ = − 10.66, *p* < 0.001) and low (t_4900_ = 3.01, *p* < 0.003) yield patches were significantly higher for self than other.

There was no significant beneficiary x environment interaction, suggesting that BRR influenced decisions regardless of beneficiary. This was supported by a post-hoc analysis showing that leaving times were significantly different between rich and poor environments for self (t_49_ = − 3.12, *p* = 0.003) and other (t_48_ = − 2.39, *p* = 0.02).

In addition, main effects of patch (t_72.95_ = 10.85, *p* < 0.001) and environment (t_69.64_ = 3.1, *p* < 0.003), were found, but not their interaction, in line with MVT predictions. Thus, despite making foraging decisions that broadly speaking conformed to the directions predicted by MVT across beneficiaries, people were more sensitive to the changes in patch yield when foraging for self than others. Importantly, examining standard deviations of leaving times in each condition suggests that these effects are not simply due to differences in decision stochasticity (see SI for details).

#### Participants forage more optimally when collecting rewards for self relative to others

The previous analysis identified differences in how people make leaving decisions when collecting rewards for others compared to collecting for themselves. But do these differences translate into optimal behaviour according to MVT? To test this, we first contrasted participants' leaving times in the different conditions against what is predicted by MVT. This analysis revealed that leaving times for all patches and environments, for both self and other, deviated significantly from the optimal solution predicted by MVT, suggesting an overstaying bias as has been observed previously in many species, including humans^26,41^ (see SI, Table S3 for details). Therefore, regardless of whether people foraged for their own rewards or for others', their behaviour was suboptimal compared to MVT predictions.

It could be possible that people showed the size of the effects of FRR and BRR according to MVT when accounting for this overstaying effect. By this, we mean whether the differences in people’s leaving times between high and low yield patches on the one hand, and between patches in the poor versus the rich environments in the other, conform to the predictions derived from MVT even though this behaviour is not strictly optimal. To determine this, we calculated the mean leaving time for each condition, and subtracted the patch (high–low yield patches) and environment (poor–rich environments) mean leaving times separately for self and other. Thus, we could examine leaving times for self and other, without the overstaying effect. We tested whether these magnitudes approximated the optimal size of these effects according to MVT, and whether this was the case when foraging for self or other. To test this, we examined whether the effects were significantly similar to the optimal prediction using formal equivalence testing. Using this approach, we found that the patch yield effect was significantly equivalent to the optimal for self (t_39_ = − 2.13, *p* =  < 0.02, 90% CI = [− 0.27, 1.10]) but not for other (t_39_ = 0.73, *p* = 0.24, 90% CI [− 2.57, − 0.47]; Fig. 2B). Indeed, FRR effect in other trials was significantly different from optimal (t_39_ = − 2.44, *p* < 0.02, 95% CI [− 2.78, − 0.26]). Consistent with MM results, a paired t-test also confirmed that FRR effect was significantly stronger in self (m = 5.06, s.d. = 2.57) than other (m = 3.12, s.d. = 3.12; t_39_ = 2.58, *p* < 0.02, 95% CI [0.42 3.46], d = 0.41). On the other hand, we found that the BRR effect was equivalent to the optimal in self (t_39_ = 1.86, *p* < 0.04, 90% CI [− 1.61, 0.20]), but not other (t_39_ = 1.01, *p* = 0.16, 90% CI [− 1.97, − 0.24]), but there was not significant difference between self (m = 1.10, s.d. = 3.40) and other (m = 0.70, s.d. = 3.24; t_39_ = 0.59, *p* = 0.56, 95% CI [− 0.99, 1.79], d = 0.09; Fig. 2D). BRR effect on other trials was significantly different from optimal (t_39_ = − 2.15, *p* < 0.04, 95% CI [− 2.14, − 0.07]). Thus, people conformed to optimal MVT predictions, once accounting for the overstaying bias, mainly when collecting rewards for themselves but not for others.

MVT also predicts that people should leave the patch when the FRR reaches the BRR of the environment. This implies that, for a given environment, foragers should leave both patches at the same FRR. Thus, a MM similar to that outlined above was performed but instead of predicting leaving times, the outcome was the FRR at the time of leaving the patch (see SI, Table S4 for full results). This analysis revealed a patch x beneficiary interaction (t_4894.46_ = 4.7, *p* < 0.001, Fig. 2C). A post-hoc analysis showed that high and low yield patches had significantly different FRR at time of leaving when collecting rewards for other (t_44.2_ = − 3.42, *p* < 0.002), but not for self (t_44.8_ = 0.04, *p* > 0.9). This result, together with the effects on leaving times, suggest a higher deviation from the optimal solution when foraging for others. The model also reveals a main effect of environment (t_59.12_ = − 3.21, *p* < 0.003), suggesting that participants adjusted their leaving times considering the BRR across conditions. Finally, an environment x patch interaction was also found (t_4888.7_ = 2.2, *p* < 0.03), but these results are likely to be driven by the great modulation that beneficiary had on patch effects. Taken together this supports the notion that people are less optimal when making foraging decisions for other due to an insensitivity to FRR.

The analysis above assumes that people were perfectly accurate in estimating the BRR. However, participants with different leaving times actually experience different BRR over time. Therefore, we calculated each participant’s long-run obtained reward rate per environment for each beneficiary, and we estimated the optimal leaving times according to those values. This approach aligns with previous studies that have considered individualised measures of reward rates to better understand decision-making processes and optimal foraging behaviour^25,26,41,59^. Participants’ average obtained BRR was significantly lower than the maximum available according to MVT across conditions (see SI, Fig. S2a). Importantly, obtained BRR was not different between self and other for neither the rich (self: m = 16.72 , s.d. = 2.62; other: m = 17.00, s.d. = 2.47; t_39_ = − 0.72, *p* = 0.48, 95% CI [− 1.05, 0.50], d = − 0.11) nor the poor (self: m = 13.87, s.d. = 2.01; other: 13.90, s.d. = 1.87; t_39_ = − 0.14, *p* = 0.89, 95% CI [− 0.51, 0.45], d = − 0.02) environments. Once optimal leaving times were adjusted according to the obtained BRR, participants still showed leaving times significantly longer than optimal for all conditions (see SI, Table S5). These results suggest that the differences between self and prosocial foraging cannot be simply attributed to a difference in the actual BRR obtained by participants.

In summary, in study 1 participants distinguished FRR and BRR in the direction predicted by MVT for both self and other. However, the effect of FRR was stronger when collecting rewards for self than others, which led participants to broadly conform with MVT predictions for self but not others.

### Study 2

In study 2, we aimed to conceptually replicate the results of study 1 whilst manipulating the average reward rate of an environment in a different manner. Specifically, rather than manipulating the BRR by changing the proportion of high vs low yield patches, we manipulated the travelling time between patches. Longer travel times reduce the BRR of an environment and make it more optimal to stay longer in patches. In study 2, the rich environment is the one where berry bushes are close to each other, while in the poor environment bushes are spread further apart. The distribution of high and low yield patches was now 50:50 across environments.

#### Sensitivity to foreground reward rate is lower when foraging for others’ vs self rewards

**Figure 3:**
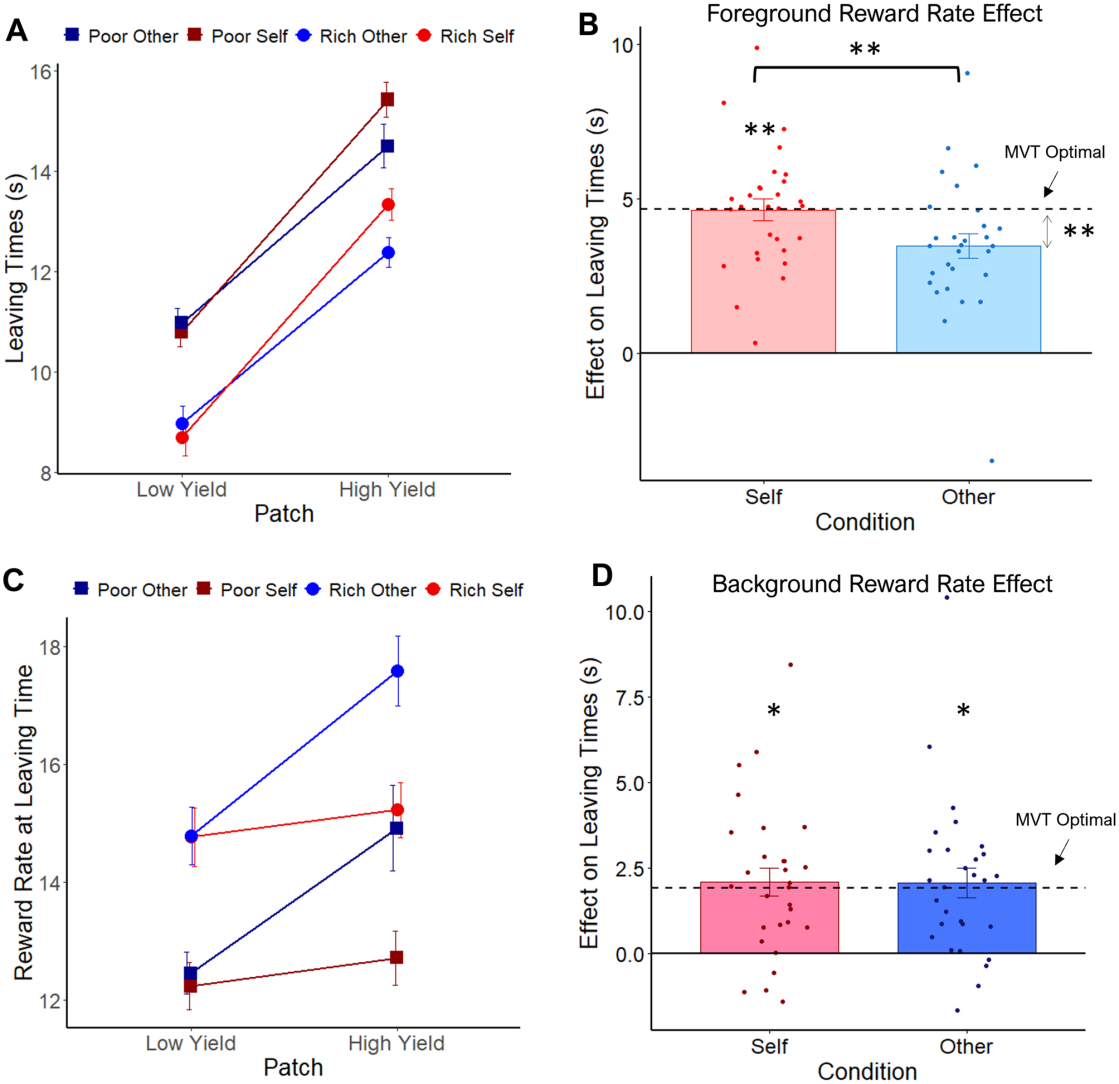
Foreground and background results of study 2. (**A**) Mean leaving times (y-axis) for each condition in study 2. Participants stayed longer in high than low yield patches (x-axis), an effect that was stronger for self than other. This was driven by a significant difference in high yield patches. Participants left the rich than the poor environment sooner across beneficiaries. Red lines correspond to self environments while blue lines are other environments, including rich (lighter circles) and poor (darker squares) environments. (**B**) Foreground reward rate effects on leaving times. Participants showed significant equivalence with optimality (dotted line) when collecting rewards for self but not others. Foreground effect deviated significantly from optimal on other trials, and it was significantly different from self. (**C**) The reward rate at leaving time was influenced by both the environment and type of patch when collecting rewards for others. For self, only the environment had an effect on patch leaving reward rates. Note according to MVT these lines should be flat, and there should be no effect of the patch yield on reward rate at leaving times. (**D**) Background reward rate effects on leaving times. Participants showed significant equivalent background reward rate sensitivity to MVT predictions for both self and other. *p < 0.05. **p < 0.01. Error bars depict s.e.m. in B and D, and within-subject s.e.m. in A and C.

To test whether the effects observed in study 1 were replicated with a different manipulation of the environment we performed the same set of analyses. Specifically, we first tested a MM on leaving times with type of patch, environment, beneficiary and their interaction as predictors. This revealed a beneficiary x patch interaction (t_7236_ = − 6.69, *p* < 0.001, Fig. 3A, see SI, Table S6 for details). A post-hoc analysis showed that the difference on leaving times between patches was significantly larger when collecting rewards for self than other (t_7238_ = − 8.48, *p* < 0.001), although the patch effect was present when collecting reward for both self and other (self: t_30.7_ = − 13.41, *p* < 0.001; other: t_30.5_ = − 9.91, *p* < 0.001). Importantly, participants stayed significantly longer in high yield patches for self than other (t_7236_ = 10.93, *p* < 0.001), but not in low yield patches (t_7237_ = − 1.04, *p* = 0.3). These results suggest that lower sensitivity to FRR when collecting rewards for others is triggered by differences in how participants perceived instantaneous reward in high yield patches. In addition, we found main effects of patch (t_34.7_ = 12.99, *p* < 0.001) and environment (t_33.7_ = 5.15, *p* < 0.001), but not their interaction, in the same direction as that predicted by the optimal solution. A post-hoc analysis revealed that participants stayed longer in the poor than the rich environment for both self (t_30_ = − 5.32, *p* < 0.001) and other (t_29.9_ = − 5.15, *p* < 0.001).

#### Participants conform broadly to optimal foraging for self but not other.

Participants stayed significantly longer than the optimal solution across conditions, displaying an overstaying bias as in study 1 (see SI, Table S7). We tested whether the FRR and BRR effects were equivalent to the optimal estimation from MVT for self and other once accounting for this bias using formal equivalence testing. Participants were optimally sensitive to variations in FRR when collecting rewards for self (t_28_ = 2.67, *p* < 0.007, 90% CI [− 0.62, 0.6]), but not for others (t_28_ = − 0.26, *p* = 0.6, 90% CI [− 1.86, − 0.50], Fig. 3B), consistent with study 1. FRR effect deviated significantly from the optimal in other trials (t_28_ = − 2.95, *p* < 0.007, 95% CI [− 0.74, 0.73]), and it was significantly lower than for self (self: m = 4.64, s.d. = 1.93, t_28_ = 3.3, *p* < 0.003, 95% CI [0.45, 1.90], d = 0.61). Finally, participants showed an optimal adjustment in their leaving times to variations in the BRR for both self (t_28_ = − 2.23, *p* < 0.02, 90% CI [− 0.51, 0.89]) and other (t_28_ = − 2.33, *p* < 0.02, 90% CI [− 0.58, 0.89], Fig. 3D), differing from study 1 which revealed optimal environment adaptation only for self. Nevertheless, and consistent with study 1, the BRR effect was not significantly different between self (m = 2.09, s.d. = 2.21) and other (m = 2.06, s.d. = 2.33; t_28_ = 0.11, *p* = 0.9, 95% CI [− 0.58, 0.65], d = 0.02).

Next, we looked at the reward rate at time of leaving. A MM with type of patch, environment, beneficiary and their interactions as predictors revealed a main effect of environment (t_42.4_ = − 5.52, *p* < 0.001), but not patch (see SI, Table S8), consistent with MVT predictions. However, and replicating study 1, a beneficiary x patch interaction was found (t_7238_ = 8.41, *p* < 0.001, Fig. 3C). A post-hoc analysis revealed that participants left high and low yield patches at a significantly different FRR for others (t_30.1_ = − 4.58, *p* < 0.001) but not self (t_30.2_ = 0.68, *p* > 0.5), different from what it is expected according to MVT.

Similar to study 1, the BRR obtained was significantly lower than the maximal available according to MVT for both environments and across beneficiaries (see SI, Fig. S2B), and they were only significantly different between self and other for the poor (self: m = 17.40, s.d. = 1.41; other: m = 16.79, s.d. = 1.48; t_28_ = 2.84, *p* < 0.01, 95% CI [0.17, 1.01], d = 0.53) but not the rich (self: m = 20.88, s.d. = 1.87; other: m = 20.55, s.d. = 2.04; t_28_ = 1.26, *p* = 0.22, 95% CI [0.86, 0.24], d = 0.24) environments. Furthermore, even if optimal leaving times are adjusted based on this participants’ specific BRR environment, participants still stayed significantly longer than predicted (see SI, Table S9).

Therefore, results in study 2 largely replicate study 1, with participants broadly conforming with MVT predictions for self but not for other. Participants were more sensitive to their own instantaneous reward compared with others’, although they seem to be similarly sensitive to the average reward in the environment across beneficiaries.

### Autistic traits are associated with reward rates effects only for self

**Figure 4:**
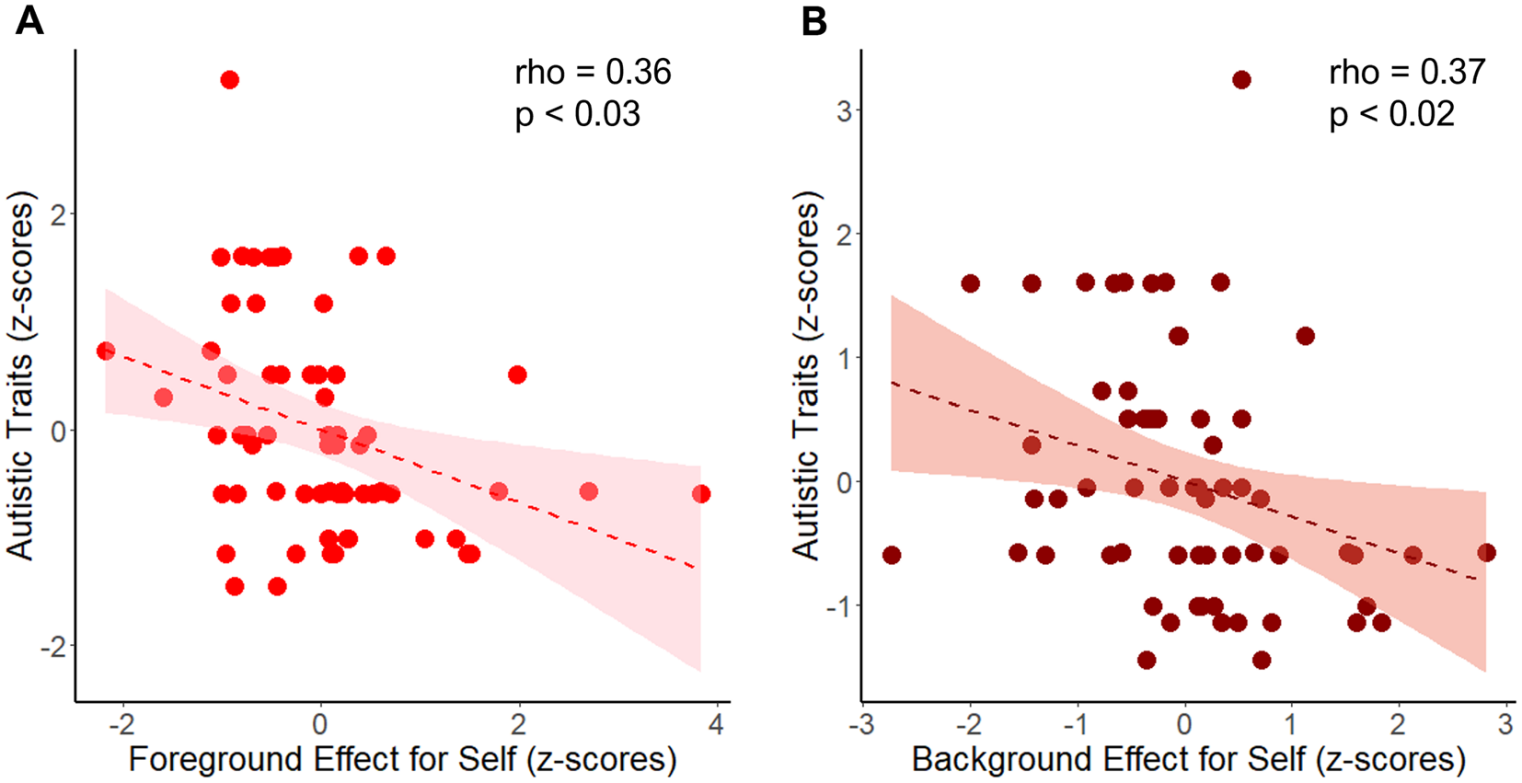
Autistic traits are associated with reward rates effects. (**A**) People who have higher scores in an autistic self-report scale (AQ-10, y-axis) are less sensitive to the foreground reward rate when making leaving decisions for self. X-axis corresponds to the difference in leaving times between high and low yield patches across environments in the self condition. (**B**) People who score high in autistic traits are less sensitive to the background reward rate for self. X-axis shows the difference in leaving times between rich and poor environments in self trials. Shaded areas show the 95% confidence interval around the slopes. Dots depict each participant.

Next, we conducted exploratory analyses to test whether psychological traits, namely autism, apathy and empathy, were associated with FRR and BRR effects, as it has been shown for other reward-based decision-making effects. We defined the FRR and BRR effects as the difference in leaving times between high and low yield patches (Figs. 2B and 3B), and between rich and poor environments (Figs. 2C and 3C), respectively, for self and other. To test for correlations, we combined the samples across studies 1 and 2. We found that AQ-10 scores were associated with FRR (rho = − 0.36, FDR corrected for multiple comparisons, *p* < 0.03) and BRR (rho = − 0.37, FDR corrected *p* < 0.02) effects only for self, but not decisions for other (FRR rho = − 0.09, *p* = 0.51; BRR rho = − 0.11, *p* = 0.39). Thus, people who had lower scores on autistic traits were more sensitive to the yield of a patch and to the environment BRR they were in when making decisions to leave when the reward was given to themselves (Fig. 4).

## Discussion

Previous research has revealed that people show a self-bias for reward, valuing their own outcomes more than others^1^. However, these studies typically do not involve dynamic and continuous decisions that more accurately reflect the type of problems solved by foragers^3,4^, where a clear optimal behaviour can be estimated. Here, we used a patch-leaving task based on the principles of MVT, a theorem of animal foraging. Participants made a series of decisions about when to leave patches with high or low reward yields, in environments with high or low average reward rates. Critically, participants collected rewards for themselves or for a stranger. Across two studies, we found that participants showed more sensitivity (a bigger change in behaviour between high and low yield patches) to the FRR when collecting rewards for themselves than for others, reflected in more optimal behaviour in responding to FRR. Intriguingly, however, participants were similarly sensitive to the BRR of the environment across beneficiaries. Finally, we found that autistic traits were linked to lower sensitivity not only to FRR but also to BRR when foraging for self. These results highlight that the self-bias may be present only for immediate rewards being obtained.

Even though participants showed suboptimal behaviour in all conditions according to MVT, they were more sensitive to instantaneous FRR when foraging for self than others, consistent with a self-bias in reward processing^1,42,45^. Strikingly, our design revealed that this pattern of behaviour closely approximated the size of the effect predicted by MVT. Furthermore, unlike other experiments, there were no costs to oneself in terms effort or loss of one’s own monetary rewards when making prosocial choices, suggesting that the self-bias for reward is not simply driven by a desire to minimise costs to ourselves, as it could be argued for some economic games^30^. Rather, our results suggest people are simply less sensitive to differences between magnitudes of rewards that others will receive compared to the same differences that we will obtain ourselves. Thus, although people can be differently sensitive to the costs we incur to obtain rewards for others^30,42,45,60^, we also do not ascribe the same value to others’ rewards.

The fact that differences were found between self and other in FRR but not BRR, is consistent with evidence showing that different types of reward processing may involve different underlying mechanisms, brain systems, and neuromodulators^26,59,61^. That is, how the brain encodes the value of a reward which is being received instantaneously by an organism may be underpinned by different mechanisms that track the average rate at which rewards are being received across time. However, our results do not provide evidence on what the specific cognitive mechanisms are underlying sensitivities to FRR and BRR. It could be possible that these reward rates, as suggested previously, are processed differently at both the neural and computational levels^62^. Future research using more sophisticated computational models of behaviour will be necessary to better unpick how people learn and change foraging behaviours.

An alternative possibility is that participants are using heuristics that guide behaviour in this, and other, foraging tasks. Heuristics are mental shortcuts or rules of thumb that simplify decision-making processes. For this study we used a design where rewards continuously and rapidly filled up a bucket at a decaying rate to avoid people using numerical or visual rules to guide decisions. Other designs used across species, with actual juice rewards^41^, or using visual stimuli representing as a number of apples in a tree^25^, have found consistent results with ours in terms of people conforming to the pattern of MVT, with an overstaying bias. Notably, one heuristic that participants might employ involves comparing time gaps between consecutive rewards. As the patch depletes, the intervals between rewards increase, and participants might decide to leave when these intervals exceed those anticipated in a new patch. This simple rule does not require participants to know the optimal leaving time explicitly but rather relies on a straightforward comparison of time intervals. Another possible heuristic is an imperfect reward rate estimation based on a fixed-duration time window. Instead of calculating the overall average reward rate throughout the entire time spent in a patch, participants might estimate the reward rate based on a recent, fixed period of time. This approach means they are considering only the most recent encounters with rewards to inform their decision. Such a heuristic could lead to less accurate estimations of the actual reward rate, as it neglects earlier rewards in the patch, but it simplifies the decision-making process by focusing on more immediate information. These heuristics could potentially explain why participants exhibit suboptimal behaviour and why their sensitivity to FRR differs when foraging for self versus others. While these heuristics do not maximise reward rate, they could account for the deviations observed in our study. Therefore, we note that we cannot say with certainty that participants are truly representing the FRR or BRR consciously and explicitly in their choices. Instead, people could be representing an approximation of these, and future research could shed light on what the rules are that govern that approximation and whether this processing depends on different or specialised implementation in the brain.

The current results also suggest that it may be possible to have specialisation for social versus self information processing in FRR^60,62,63^. The fact that this online tracking of instantaneous rewards was different between self and other might indicate that different motivational factors or biases can influence this rate of reward processing^34,35,64^ although they were beyond the scope of this study to precisely specify. Although our results cannot rule out the possibility that the average BRR is open to similar biases, we found less evidence of this, suggesting that the tracking of average reward rates over time is not associated with motivational factors or biases. Thus, BRR might be linked to capturing the properties of the task to succeed and optimise reward intake regardless of the recipient, in a domain-general, non-specialised process^62,65–72^ Future research should therefore examine whether manipulating systems in the brain that process average reward rates or opportunity costs influences both self and other reward processing in similar or different ways.

While our results indicate variations in foraging behaviour when collecting rewards for oneself versus others, the distinction from foraging when collecting rewards for no one remains unknown as our design lacked a control condition with these characteristics. We opted not to include such a condition as our primary aim was to compare the optimality of foraging behaviour when collecting rewards for others versus oneself, making a no-reward condition outside the scope of this study. Additionally, in natural settings, organisms typically forage resources that will be used, making a foraging-for-no-one condition less ecologically relevant^73^. However, as mentioned earlier, the tracking of BRR may encode the structural properties of the task, potentially independent of the value of the reward. Moreover, while our results demonstrate that individuals are more sensitive to FRR for themselves than for others, we cannot determine whether rewards for others are prioritized over no reward at all in a foraging task. Although previous studies suggest this possibility^35,36,74^, it has not been specifically tested in a foraging context. Therefore, future research could explore these questions by comparing different conditions to gain a deeper understanding of the underlying mechanisms.

The aim of this study was to examine whether foraging was optimal when comparing self foraging versus foraging for an anonymous other. This allowed us to examine in the most stripped down of situations whether people’s basic foraging behaviour was different when there was no possible influence of reciprocity or reputation building on people’s behaviour. It is important to note that, from an energy intake standpoint, there may not be an inherent conformity to MVT principles when foraging prosocially for a stranger, as individuals do not derive energy directly from this behaviour. However, this lack of direct energy gain is also true in this economic game context where participants were foraging for their own monetary benefit. Consistently, even though people, generally, followed MVT principles, they were more optimal when collecting resources for themselves than for someone else.

Foraging for others, such as strangers, could emerge as a hidden rule based on mutual reciprocity^7,75^. It has been previously proposed that social interests and values are based on past learning contingencies on reciprocity and cooperation, with sharing resources being rewarded and the opposite being punished^76–78^. However, given that prosocial actions do not have a direct consequence to the agent, and in reciprocal motivation the direct benefit is delayed and uncertain, people might show more variability in their sensitivities to others’ rewards compared with self. Future research could explore this aspect further, considering the complexities of human cooperation, social learning, and the presence of hidden social rules influencing behaviours within a group. Addressing these aspects could contribute to a more comprehensive understanding of the observed behaviours in our experiments.

As noted above, from a basic biological perspective, foraging typically serves the individual's immediate needs for sustenance and survival. However, foraging for others can vary depending on the relationship with the recipient. For example, individuals may show different behaviours when foraging for kin or members of their own social group compared to foraging for strangers^12,73^. People are generally more motivated to collect rewards for close rather than distant others^32,33,79^. Thus, motivational factors might influence foraging for others and our results should be interpreted within the context of foraging for anonymous other individuals compared to oneself^14,30^. Unlike reciprocal altruism, where the expectation of a future benefit might motivate prosocial behaviour, foraging for anonymous others lacks this direct or delayed personal benefit. Future studies could further explore how the nature of the relationship influences foraging behaviour and the underlying motivations.

The self-bias has been shown across many domains of behaviour^80^. People are faster to learn about rewards we can obtain^35^, about what stimuli belong to us^74,80^, and work harder to obtain rewards for ourselves^45^. While a powerful influence on behaviour, in most of these examples, there is no optimal behaviour. Doing something more quickly, or something being more salient, does not necessarily make the behaviour more optimal. For example, people exert more effort than is necessary when trying to obtain rewards for self but not for others^45^. In very few studies it is possible to test whether a behaviour is optimal. Foraging-based tasks and MVT have the distinct advantage that an optimal behaviour is defined by the environment^3^. Thus, here it was possible to identify whether people were more optimally changing their behaviour when making choices that affected the rewards obtained by self and other. This offered the possibility to directly test whether the self-bias was present, and whether it was optimal. Such findings offer promise for demonstrating how foraging-based paradigms can shed new light on long unanswered questions in psychological and decision neuroscience research^3,4,81^.

The results of this study highlight the utility of using behavioural ecology-based approaches to examine psychological questions beyond typical prosocial motivational tasks^3,4,41,82^. Questions often examined in social psychology and neuroscience, such as how sensitive we are to self and others’ reward, can be probed and better understood when considering different reward rates and the optimality that is afforded by using MVT based tasks. Still, the conclusions derived from using foraging tasks in cognitive sciences should be taken cautiously, as the resources that are collected from these kinds of tasks are very different from the ones animals face in natural environments. Therefore, these experiments allow us to understand basic determinants and processes of foraging, such as decision-making in the face of changing reward rates, in a dynamic setting that emulates ecological choices, acknowledging the inherent differences between experimental setups and natural activities.

We found here that people are differently sensitive to FRR but not BRR, and we have interpreted this as a difference in motivational value of FRR, but not BRR, which might be captured as a structure property of the task. However, an alternative interpretation for high sensitivity to the FRR when foraging for self could be higher learning rates when collecting self rewards^25,83^. Previous work has shown in binary choice paradigms that people have higher learning rates for self than other and that learning for self and other may depend on distinct systems in the brain^35^. While not directly explored in our study, variations in learning rates could suggest that distinct brain mechanisms might underlie self and other foraging behaviours. Future research could investigate how existing reinforcement learning models might effectively capture patch foraging behaviour, and how this is different when collecting resources for oneself or for others.

Learning could have also influenced optimal behaviour in this task^83,84^. For instance, it is not clear whether more time in each environment could have made people’s behaviour closer to MVT predictions. In order to estimate accurately the BRR of the environment, agents need to learn from sampling its patches. Even though participants spent several minutes in each environment, it is still an open question whether making this sampling longer could change their behaviour. However, from this particular task design, it is unlikely that to be the case. First, even though participants spent 10 min longer in the rich and the poor environments in study 2, they still show overstaying. Second, across studies, participants displayed suboptimal leaving times for each patch according to their own obtained BRR, which makes unlikely that this suboptimality is only based in sampling patches. Finally, given the nature of our task, in which participants must perform a simple motor command each time, making it too long could have produced boredom in participants, which might cause even worse performance as suggested by previous studies^85,86^. Therefore, future research could examine how patch sampling and learning rate can affect foraging performance using a different approach than our foraging task, which was mainly focused on differences between self and prosocial foraging.

We found that autistic traits were linked to sensitivity to both FRR and BRR but only for self. This might be surprising given the social motivation hypothesis of autism, which argues that that social disruptions in autism are derived from a reduction in how rewarding social stimuli are perceived^46,87^. However, in the current study the reward is not social *in nature* (as in a social interaction, e.g., happy faces), and the rewarding experience is not necessarily felt by the agent but by a third-party. In this sense our results are consistent with others showing that higher levels of autistic traits are not necessarily linked to lower levels of empathy or lower willingness to act prosocially^88,89^. Rather, higher levels of autistic traits might be associated with generalised changes in how the brain processes rewards ^43,47,90^. Although further work is needed to explain the mechanisms behind our results, it is notable that higher levels of autistic traits are associated with differences in non-social processing that influence decision-making. Thus, dissociating self from social-specific processes in autism may be crucial.

When to leave a rewarding activity for another is a key decision problem in multiple situations. Here, using foraging theories, we designed a task where participants made these kinds of decisions to obtain rewards either for themselves or another person. Our results reveal that people are more optimal when foraging for self than for a stranger, suggesting the self-bias may have been an advantageous adaptation.

## Data availability

All data and scripts used for main analysis and figures can be found here https://osf.io/qmpw7/?view_only=55383fa079d04acc9838d28812621f32.

## Supplementary Information

Supplementary Information.
