# Supplementary material for: A reward self-bias leads to more optimal foraging for ourselves than others
Source: Sci Rep. 2024 Nov 5;14:26845. doi: 10.1038/s41598-024-69452-x (PMC11538449; doi:10.1038/s41598-024-69452-x)
Supplement: Supplementary file 1 — Supplementary Information. [file 41598_2024_69452_MOESM1_ESM.docx]

**Supplementary Information for “A reward self-bias leads to more optimal foraging for ourselves than others”**

Luis Sebastian Contreras-Huerta^a,b,c,d,e*^, M. Andrea Pisauro^c,d^, Svenja Kuechenhoff^b^, Arno Gekiere^a^, Campbell Le Heron^f^, Patricia L. Lockwood^a,b,c,d,h^ and Matthew A. J. Apps^a,b,c,d,h*^

^a^ Department of Experimental Psychology, University of Oxford, Oxford, Oxford OX1 3PH, UK

^b^ Wellcome Centre for Integrative Neuroimaging, University of Oxford, FMRIB, John Radcliffe Hospital, Oxford OX3 9DU, UK

^c^ Centre for Human Brain Health, School of Psychology, University of Birmingham, Birmingham B15 2TT, UK

^d^ Institute for Mental Health, School of Psychology, University of Birmingham, Birmingham B15 2TT, UK

^f^ Center for Social and Cognitive Neuroscience (CSCN), School of Psychology, Universidad Adolfo Ibáñez, Viña del Mar, Chile

^g^ Department of Medicine, University of Otago, Christchurch, New Zealand

^h^ Christ Church, University of Oxford, Oxford OX1 1DP, UK

***Correspondence:** Luis Sebastian Contreras-Huerta, [l.contrerashuerta@bham.ac.uk](mailto:l.contrerashuerta@bham.ac.uk); Matthew A.J. Apps, [m.a.j.apps@bham.ac.uk](mailto:m.a.j.apps@bham.ac.uk).

**Supplementary Methods**

*Random assignment procedure*

In the self-other foraging task (SOFT), participants collected rewards for either themselves or for another anonymous person. We followed a rigorous protocol to ensure they believed that the task outcomes affected real people [1,2]. Before participants performed the task, they were told that two participants were taking part in the experiment, who would be randomly assigned to one of two decision-making tasks, which have different characteristics – one would involve collecting rewards as bonus (i.e. the SOFT), and one would involve making choices on preferred items. The tasks were assigned randomly by selecting one of two balls from a box. In reality, however, all participants performed the SOFT, as the other participant was a confederate.

In the assignment procedure, participants were inside the experimental room with the experimenter who explained the protocol. Then, a second experimenter arrived at the experimental room with the confederate standing on opposite sides of the participant in a semi-opened door, obscured from each other’s view. Both the participant and the confederate were instructed not to talk out loud, and they were handed a rubber glove to prevent any feature that could influence decisions from being seen. Both participants and confederates were told to place their gloved hand in front of the door and to wave to one another in order to make sure that they were aware of the other person’s presence. Then they proceeded to take a ball from a box held by one of the experimenters. The order in which they took the ball was decided by tossing a coin. After this, the second experimenter and the confederate left, and participants were informed that they would play the task associated with collecting rewards, while the other participant would perform the task based on preferences.

During the explanation of the SOFT, participants were told that they would not collect rewards only for themselves, but also for the other participant, so they could also get a bonus for their participation. They were told that the rewards that they collected would be given to the other participant as a bonus, but the other participant would not know where that bonus had come from. They would simply be told that the bonus was an extra payment for their participation, without saying that it was collected by other participants. This procedure ensured that their choices in the task were made anonymously, and that they would not meet or interact with the other participant. These instructions aimed to minimise concerns about reputation, social desirability and reciprocity as these factors could influence their social behaviour outside of their desire to be prosocial [3].

At the end of the session, participants were asked debriefing questions, to detect whether they had any doubts about the deception throughout the experiment. Crucially, these questions were subtle, and none of them directly ask the reliability of the procedure, avoiding inducing concerns about the veracity of the manipulation.

*Social Foraging Task Instructions*

The following instructions correspond to the ones given in study 2. For study 1, the subsection *Types of Farms* was modified accordingly to its specific manipulation.

Introduction

In this game, you will be visiting farms where you are going to be collecting berries from “virtual bushes”. You will be collecting raspberries for yourself, and blueberries for the other participant. In this experiment the berries will hold monetary value. The more raspberries you collect, the more money you will earn for yourself. Similarly, the more blueberries you collect the more money you will earn for the other participant. The other participant won’t know that you are collecting berries/money for them. What you earn for them today will be added to their overall payment for their participation in this experiment, without telling them that a bonus has been added. Furthermore, what you collect for yourself does not affect in any way what you collect for the other participant, and vice versa. Therefore, your participation will be anonymous and confidential, and what you collect for you and the other participant are independent from each other.

You will collect the berries by pressing the space bar while in a field (with the bushes). Here you can see the field, visualised by a basket. The colour bar represents the volume of berries you have collected so far in the field. The pink bar represents raspberries collected for you, and the blue bar represents the blueberries collected for the other person. Also, the recipient of the berries will be indicated at the top of the screen. As you spend time in the field, the bar will go up. But, the longer you spend in the field, the slower it will go up – this means the rate of berry collection reduces the longer you stay in the field.

Therefore, at some point you will want to leave this field to travel to a new one. When you arrive at the next field the berry rate goes back to the highest (for that field). However, it takes time to travel between fields and during that time you cannot collect any berries at all.

There are an infinite number of fields available to you – but a limited amount of time.

Your job is to decide when to leave each field. If you leave each field very early or very late you won’t collect as much over the whole experiment.

Any questions so far?

Types of fields

There are 2 types of fields. One type is a “poor” field, which doesn’t have many bushes. This means the amount of collected berries increases at a slower rate and you get less berries over time when compared to the other field. The second type is a “good” field with a high number of bushes. In this type, the amount of collected berries will increase quickly, so you can earn more in a set amount of time. You will get a chance to see these different field types when we do a practice in a moment.

Types of Farm

The fields will be arranged in 2 types of farms. You will spend 5 minutes on each farm. In the gold farm, the fields are very close to each other, so the travel time between fields is short. On the other hand, in the green farm, the fields are further from each other, having longer travel times between fields.

This means that when you are in a gold farm and choose to leave a field, you will reach the next field faster than in the green farm. However, both farms have the same distribution of rich and poor fields, which is 50% each. That means that when you choose to leave a field, regardless if you are in a gold or green farm, the chance that the next field is a good or bad one is 50%. The only difference between gold and green farms is that the time between fields in the gold one is shorter than in the green one.

You will be notified when entering a new farm through a message saying, ‘You have entered a new farm, and the change in frame colour (gold or green). Also, there will be farms where you collect raspberries for yourself and farms where you collect blueberries for the other person. This will be indicated at the top of the screen once you enter in the new farm (in pink for raspberries and blue for blueberries). Finally, the travel time will be also indicated with a clock, with a higher time in the green farm, and a shorter time in the gold one.

Summary

So, overall, in half of the farms you will be collecting raspberries for yourself and in the other half you will be collecting blueberries for the other participant. Within these farms, you need to decide when to leave each field.

You need to take into account:

• How long it takes to walk between fields (illustrated by the circle in the right)

• What the rate of berry return is in the current field.

• Whether you’re on a gold or green farm – i.e. what is the travel time to reach the next field.

• The limited time (5 minutes) you have within each farm.

Remember, berries will be converted into money at the end of the experiment, with raspberries being for yourself and blueberries for the other person!

*Optimal predictions using the Marginal Value Theorem (MVT)*

MVT formalises the patch-leaving problem of an agent foraging for rewards in a in an environment that has different foreground and background reward rates, which depend on the energetic costs of foraging [4,5]. Thus, MVT provides a solution that maximises the energy intake of the agent given the environment. MVT assumes that patches deplete over time, which is a natural property of many resources (the more that is consumed the less there is available and thus the slower your intake will be). **Figure 1b** in the main text illustrates how the high (purple) and low (orange line) yield patches decrease with time following the patch reward function (*g(T)*) in **equation 1**. To calculate optimal leaving times, MVT uses the following formula for a patch type *i* to compute the background reward rate of the environment *R*:

$$R= \frac{P_{i}g_{i}{(T}_{i})- k_{i}}{d_{i}+ P_{i}T_{i}} (S1)$$

Where *P* is the probability to encounter patch *i*, that has a patch reward function of *g(T)*, *k* is the energetic cost of foraging, *d* is the travel time, and *T* is the patch residency time. Note that R indicates the reward rate of the environment in energy units. Thus, the right part of the S1 equation corresponds to the patch return rate given the travel time, the time spent in each patch and the encounters with them. The BRR is maximised when its derivative, considering the time, is equal zero, such that R’(t) = 0.

Differentiating this formula relative to time and rearranging its elements, shows that R is maximised when leaving times meet the following:

$${g'}_{i}{(T}_{i})= \frac{P_{i}g_{i}{(T}_{i})}{d_{i}+ P_{i}T_{i}} (S2)$$

Where the left part of the equation corresponds to the foreground reward rate, while the right part is the background reward rate. Notice that the equation above does not contain the parameter k, as in the experiments reported here foraging did not have energetic costs associated. Therefore, according to MVT, the optimal leaving time is when instantaneous foreground reward rate equals the average background reward rate of the environment. Calculating leaving times allows to test for optimal behavioural choices considering the different elements of the MVT.

Using MVT, studies 1 and 2 were designed in such a way that optimal leaving times were defined *a priori*, according to the parameters used in each experiment. In **Figure 1b**, green dots correspond to the leaving times for studies 1 (left) and 2 (right) in the poor environment, while gold dots are for the gold environment. Green and gold dashed lines correspond to the reward rate in which it is optimal to leave the poor and the rich environments respectively. Thus, longer patch residency time is predicted for high than low yield patches regardless of the environment, as the former foreground reward rate reaches the background reward rate later than the latter. This effect (difference in optimal leaving times between high and low yield patches) was set at five seconds across studies 1 and 2. Furthermore, MVT predicts a longer residency time in the poor, green environment than in the rich, gold environment irrespective of the patch. The environment effect (difference in optimal leaving times between poor and rich environment) was set in two seconds across studies. Importantly, notice that the reward rate at the time of leaving is the same for high and low yield patches within an environment, as at the optimal time of leaving a patch its reward rate equals the reward rate of the environment.

**Supplementary Results**

*ANOVA on standard deviations of participants’ leaving times per condition*

To test whether the patch effect on leaving times between self and other (mixed-model reported in the **Results** section in the main text) was partially due to differences in decision stochasticity, we looked at the standard deviations of participants’ leaving times. We calculated, for each of our eight conditions (high/low yield patch x poor/rich environment x self/other) the standard deviation of leaving times. We took standard deviations as a proxy for stochasticity of leaving decisions. Thus, in study 1 and 2, we performed a 3-way repeated measures ANOVA on leaving times standard deviations, with three factors having two levels each: Patch (high and low), Environment (rich and poor), and Beneficiary (self and other).

For study 1, results revealed no main effects or interactions with standard deviations, suggesting that people have similar variability in their leaving times across conditions, and specifically, between patch x beneficiary (F(39, 39.5) = 0.56, p = 0.46). In study 2, on the other hand, there was only a main effect of beneficiary, such that standard deviations were higher for other (M = 3.06, SEM = 0.2) than self (M = 2.65, SEM = 0.18; F(28, 21.16) = 12.6, p < 0.001). Thus, people showed more variability in their leaving decisions when collecting rewards for others than for self. Nevertheless, and crucially, the patch x beneficiary interaction was not significant (F(28, 15.52) = 0.78, p = 0.38), suggesting that variability in leaving times did not influence the patch effect reported in the main text. Note that neither in the mixed-models of study 1 nor in study 2 did we find a main effect of beneficiary on leaving times (**Tables S2** and **S6** below). This suggests that the effect found on standard deviations in study 2 relates to factors different from our experimental manipulations.

*Differences in total reward accrued between self and other*

The main behavioural indexes in the social foraging task were leaving times and reward rate at time of leaving a patch. However, differences in leaving times do not necessarily translate into significant differences in total reward accrued, making the latter index less sensitive. Given that participants overstayed in patches, the reward gain over time is minimal, i.e. higher differences in reward occur early in the patch as it is evident in Fig, 1B of the main text. Thus, people in study 1 collected slightly more rewards for others (18,713 arbitrary units au) than self (18,502 au), but this difference was not significant (Wilcoxon Signed-Rank Test, z = -1.48, p = 0.14). In contrast, participants in study 2 collected significantly more rewards in total for self (33,657 au) than others (32,914 au; z = - 2.2, p < 0.03) albeit weakly, suggesting that differences in optimality in this study impacted the reward accrued by participants for each condition. Nevertheless, these results must be taken with caution given the reasons above.

**Supplementary Tables**

Abbreviations used in tables: *P = Patch; E = Environment; B = Beneficiary; b = beta estimate; SE = Standard Error; SD = Standard Deviation; df = degrees of freedom.*

*Table S1. Random-slopes model outperforms a model with only random intercept*

| Mixed-Model on leaving times | AIC | Log-likelihood |
| --- | --- | --- |
| Study 1 - Only random intercept | 29747 | -14864 |
| Study 1 - Random intercept + random slopes | 29393 | -14682 |
| Study 2 - Only random intercept | 38408 | -19194 |
| Study 2 - Random intercept + random slopes | 37408 | -18689 |

*Table S2. Results of the mixed-model on leaving times in study 1*

| Effect | b | SE | df | t | p |
| --- | --- | --- | --- | --- | --- |
| P | 4.90 | 0.45 | 72.95 | 10.85 | < 0.01 |
| E | 1.44 | 0.46 | 69.64 | 3.10 | < 0.01 |
| B | -0.46 | 0.33 | 4900.74 | -1.42 | 0.16 |
| P*E | -0.24 | 0.39 | 4888.80 | -0.62 | 0.54 |
| P*B | -1.37 | 0.39 | 4898.84 | -3.54 | < 0.01 |
| E*B | -0.24 | 0.39 | 4897.42 | -0.62 | 0.54 |
| P*E*B | -0.15 | 0.54 | 4891.66 | -0.28 | 0.78 |

*Table S3. Overstay bias in all conditions in study 1. Results correspond to one sample t-tests against the optimal solution predicted by MVT.*

| Condition | Mean LT (s) | SD (s) | Optimal (s) | t | p |
| --- | --- | --- | --- | --- | --- |
| Low P, Rich E, Self | 12.77 | 8.79 | 5.15 | 5.49 | < 0.01 |
| Low P, Rich E, Other | 12.12 | 6.88 | 5.15 | 6.41 | < 0.01 |
| High P, Rich E, Self | 17.94 | 8.68 | 9.79 | 5.94 | < 0.01 |
| High P, Rich E, Other | 15.51 | 6.28 | 9.79 | 5.76 | < 0.01 |
| Low P, Poor E, Self | 13.98 | 8.33 | 6.95 | 5.34 | < 0.01 |
| Low P, Poor E, Other | 13.09 | 6.9 | 6.95 | 5.62 | < 0.01 |
| High P, Poor E, Self | 18.94 | 8.82 | 11.6 | 5.26 | < 0.01 |
| High P, Poor E, Other | 15.95 | 6.06 | 11.6 | 4.54 | < 0.01 |

*Table S4. Results of the mixed-model on reward rates at the time of patch leaving in study 1*

| Effect | b | SE | df | t | p |
| --- | --- | --- | --- | --- | --- |
| P | -0.47 | 0.60 | 58.59 | -0.78 | 0.44 |
| E | -1.88 | 0.59 | 59.12 | -3.21 | < 0.01 |
| B | 0.27 | 0.35 | 4894.99 | 0.79 | 0.44 |
| P*E | 0.90 | 0.41 | 4888.69 | 2.20 | 0.03 |
| P*B | 1.93 | 0.41 | 4894.46 | 4.7 | < 0.01 |
| E*B | 0.52 | 0.41 | 4893.62 | 1.27 | 0.20 |
| P*E*B | 0.04 | 0.57 | 4890.11 | 0.07 | 0.95 |

*Table S5. Study 1 results of one sample t-tests on the difference between mean participants’ leaving times per condition and their specific MVT prediction according to their experienced BRR*

| Condition | Mean (s) | SD (s) | t | p |
| --- | --- | --- | --- | --- |
| Low P, Rich E, Self | 6.05 | 7.34 | 5.21 | < 0.01 |
| Low P, Rich E, Other | 5.58 | 5.94 | 5.94 | < 0.01 |
| High P, Rich E, Self | 6.58 | 7.19 | 5.79 | < 0.01 |
| High P, Rich E, Other | 4.32 | 5.40 | 5.06 | < 0.01 |
| Low P, Poor E, Self | 5.60 | 6.95 | 5.09 | < 0.01 |
| Low P, Poor E, Other | 4.73 | 5.93 | 5.05 | < 0.01 |
| High P, Poor E, Self | 5.92 | 7.50 | 5.00 | < 0.01 |
| High P, Poor E, Other | 2.95 | 5.36 | 3.48 | < 0.01 |

*Table S6. Results of the mixed-model on leaving times in study 2*

| Effect | b | SE | df | t | p |
| --- | --- | --- | --- | --- | --- |
| P | 4.64 | 0.36 | 34.66 | 12.99 | < 0.01 |
| E | 2.12 | 0.41 | 33.74 | 5.15 | <0.01 |
| B | 0.16 | 0.13 | 7235 | 1.23 | 0.22 |
| P*E | < 0.01 | 0.20 | 7235 | 0.01 | 0.99 |
| P*B | -1.26 | 0.19 | 7236 | -6.69 | < 0.01 |
| E*B | -0.12 | 0.20 | 7236 | -0.58 | 0.56 |
| P*E*B | 0.09 | 0.29 | 7234 | 0.32 | 0.75 |

*Table S7. Overstay bias in all conditions in study 2. Results correspond to one sample t-tests against the optimal solution predicted by MVT.*

| Condition | Mean LT (s) | SD (s) | Optimal (s) | t | p |
| --- | --- | --- | --- | --- | --- |
| Low P, Rich E, Self | 8.70 | 3.92 | 3.40 | 7.28 | < 0.01 |
| Low P, Rich E, Other | 8.98 | 4.44 | 3.40 | 6.77 | < 0.01 |
| High P, Rich E, Self | 13.34 | 4.33 | 8.04 | 6.59 | < 0.01 |
| High P, Rich E, Other | 12.39 | 4.99 | 8.04 | 4.69 | < 0.01 |
| Low P, Poor E, Self | 10.80 | 4.67 | 5.30 | 6.36 | < 0.01 |
| Low P, Poor E, Other | 10.98 | 5.20 | 5.30 | 5.88 | < 0.01 |
| High P, Poor E, Self | 15.43 | 5.08 | 9.95 | 5.81 | < 0.01 |
| High P, Poor E, Other | 14.50 | 6.05 | 9.95 | 4.06 | < 0.01 |

*Table S8. Results of the mixed-model on reward rates at the time of patch leaving in study 2*

| Effect | b | SE | df | t | p |
| --- | --- | --- | --- | --- | --- |
| P | 0.42 | 0.63 | 33.74 | 0.66 | 0.51 |
| E | -2.59 | 0.47 | 42.39 | -5.53 | < 0.01 |
| B | 0.10 | 0.21 | 7237 | 0.45 | 0.65 |
| P*E | < 0.01 | 0.33 | 7236 | < 0.01 | 0.99 |
| P*B | 2.53 | 0.30 | 7238 | 8.41 | < 0.01 |
| E*B | 0.27 | 0.32 | 7239 | 0.84 | 0.40 |
| P*E*B | -0.30 | 0.46 | 7236 | -0.66 | 0.51 |

| Condition | Mean (s) | SD (s) | t | p |
| --- | --- | --- | --- | --- |
| Low P, Rich E, Self | 4.09 | 3.16 | 6.99 | < 0.01 |
| Low P, Rich E, Other | 4.22 | 3.55 | 6.40 | < 0.01 |
| High P, Rich E, Self | 4.09 | 3.64 | 6.05 | < 0.01 |
| High P, Rich E, Other | 2.99 | 4.20 | 3.83 | < 0.01 |
| Low P, Poor E, Self | 4.56 | 4.08 | 6.02 | < 0.01 |
| Low P, Poor E, Other | 4.40 | 4.52 | 5.24 | < 0.01 |
| High P, Poor E, Self | 4.55 | 4.53 | 5.40 | < 0.01 |
| High P, Poor E, Other | 3.28 | 5.47 | 3.23 | < 0.01 |

*Table S9. Study 2 results of one sample t-tests on the difference between mean participants’ leaving times per condition and their specific MVT prediction according to their experienced BRR*

**
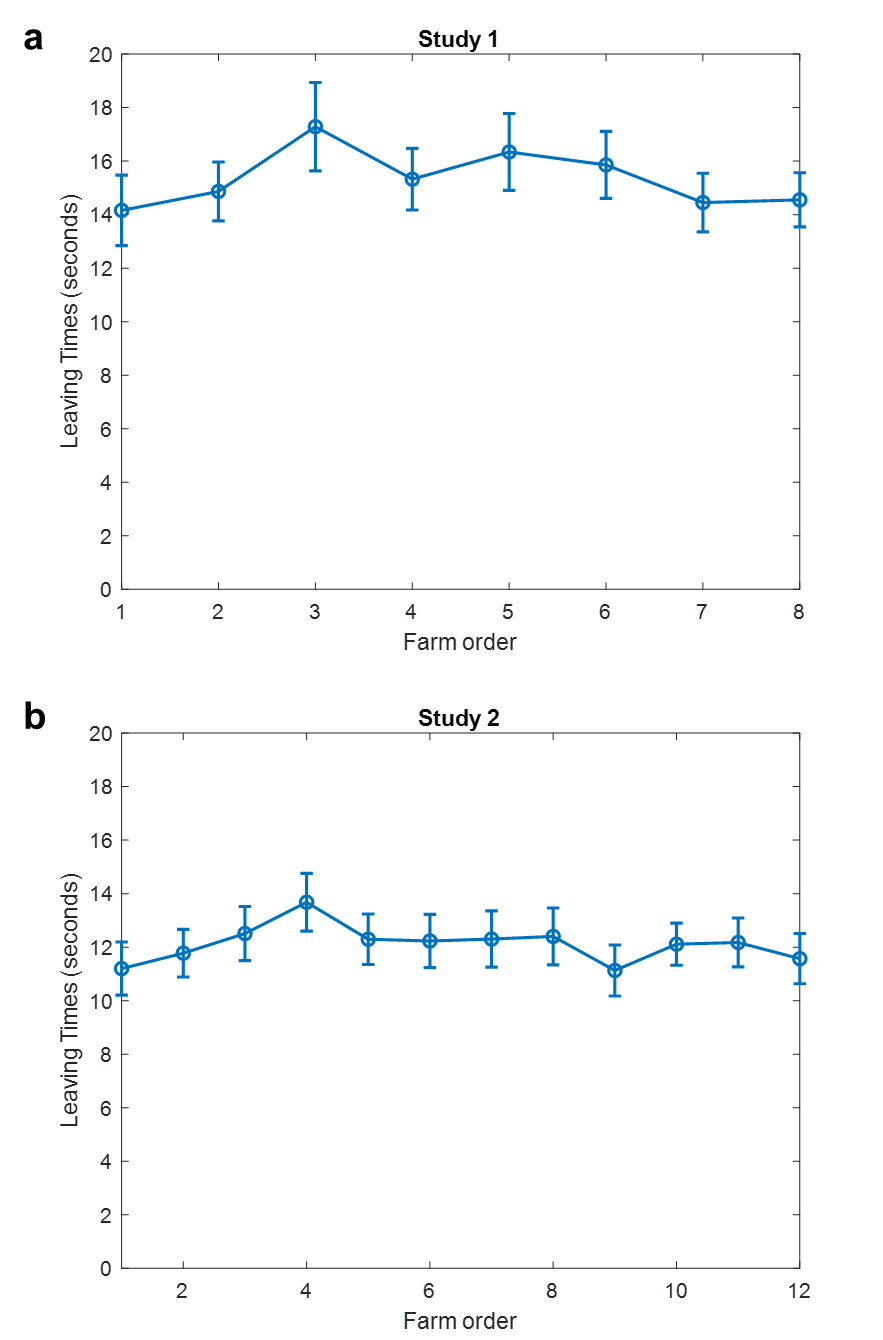
Supplementary Figures**

**Figure S1.** Average leaving times of participants are depicted according to the sequence of farm presentations. The x-axis represents the order in which farms were presented, while the y-axis shows the average leaving times of participants, with error bars indicating standard errors. Panel a corresponds to the findings of study 1, which consisted of two blocks, while panel b represents the results of study 2, comprising three blocks. Each block involved the random presentation of the four types of farms.

**
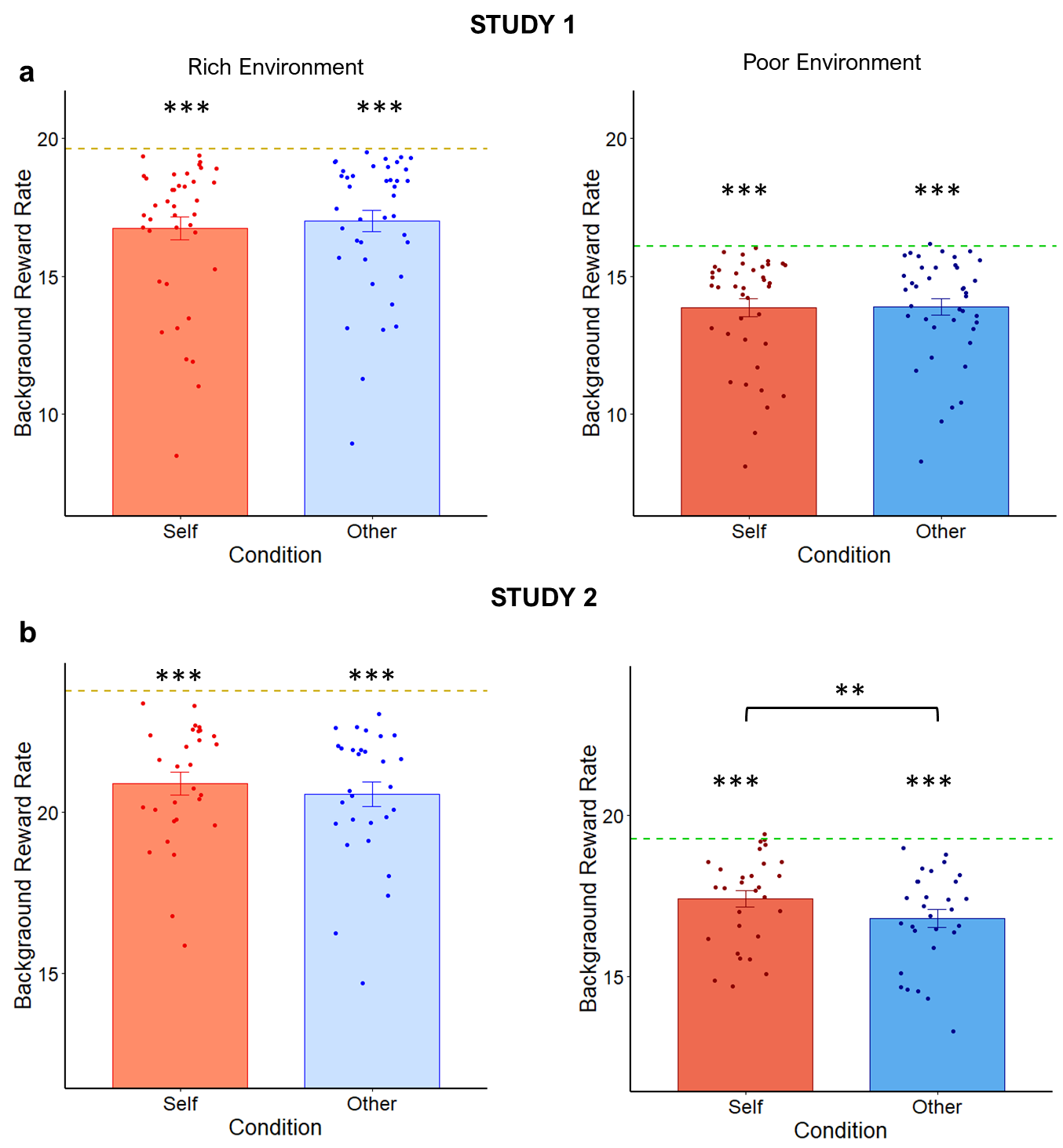
**

**Figure S2.** Mean obtained long-run background reward rates (y-axis) in the rich and poor environments for self and other in studies 1 **(a)** and 2 **(b)**. Experienced background reward rates were significantly lower than the actual background reward rate in the rich (gold dotted line, left plots) and in the poor (green dotted line, right plots) environments (one-sample t-tests, p < 0.001). Obtained background reward rates were not significantly different between self and other in study 1 (paired t-test, p >0.45 in both environments). However, in study 2, participants experienced significantly higher background reward for self than other in the poor environment (paired t-test). For the rich environment there was not a significant difference between self and other in study 2. *** p < 0.001. ** p < 0.01. Error bars depict SEM.

**References**

1. Lockwood PL, Hamonet M, Zhang SH, Ratnavel A, Salmony FU, Husain M, Apps MAJ. 2017 Prosocial apathy for helping others when effort is required. *Nat. Hum. Behav.* **1**, 0131. (doi:10.1038/s41562-017-0131)

2. Crockett MJ, Kurth-Nelson Z, Siegel JZ, Dayan P, Dolan RJ. 2014 Harm to others outweighs harm to self in moral decision making. *Proc. Natl. Acad. Sci.* **111**, 173201–17325. (doi:10.1073/pnas.1424572112)

3. Rilling JK, Sanfey AG. 2011 The neurobiology of social decision-making. *Annu. Rev. Psychol.* **62**, 23–48. (doi:10.1016/j.conb.2008.06.003)

4. Charnov EL. 1976 Optimal foraging, the marginal value theorem. *Theor. Popul. Biol.* **9**, 129–136.

5. Gabay AS, Apps MAJ. 2020 Foraging optimally in social neuroscience: computations and methodological considerations. *Soc. Cogn. Affect. Neurosci.* , 1–13. (doi:10.1093/scan/nsaa037)
